# Recycled GFRP Aggregate Concrete Considering Aggregate Grading: Compressive Behavior and Stress–Strain Modeling

**DOI:** 10.3390/polym14030581

**Published:** 2022-01-31

**Authors:** Yingwu Zhou, Yitao Weng, Limiao Li, Biao Hu, Xiaoxu Huang, Zhongfeng Zhu

**Affiliations:** 1 College of Civil and Transportation Engineering, Shenzhen University, Shenzhen 518060, China; ywzhou@szu.edu.cn (Y.Z.); wengyitao2019@email.szu.edu.cn (Y.W.); 1900471002@email.szu.edu.cn (L.L.); xxhruby@szu.edu.cn (X.H.); zhongfeng.zhu@szu.edu.cn (Z.Z.); 2 Guangdong Provincial Key Laboratory of Durability for Marine Civil Engineering, Shenzhen University, Shenzhen 518060, China; 3 Key Laboratory for Resilient Infrastructures of Coastal Cities, Shenzhen University, Ministry of Education, Shenzhen 518060, China

**Keywords:** FRP waste, recycling FRP, recycled FRP aggregate concrete, stress–strain behavior, uniaxial compressive test

## Abstract

Fiber-reinforced polymer (FRP) composites have been used in various industries, thus a large amount of FRP wastes have been generated due to the out-of-service of FRP products. Recycling FRP wastes into coarse aggregates to replace natural coarse aggregates (NCA) to form the recycled FRP aggregate concrete (RFAC) is a potential approach to dispose of huge quantities of FRP wastes with low environmental impact. In this paper, waste glass FRP (GFRP) bars were cut into particles of 12 sizes to enable the grading of recycled FRP aggregate (RFA) as similar as possible to that of NAC. The influence of different RFA volume replacement ratios (0%, 30%, 50%, 70%, 100%) on the compressive performance of RFAC was investigated based on uniaxial compression tests of 15 standard cylinders. The results showed that the failure mode of RFAC was different from that of NAC. As the RFA replacement ratio increased, the compressive strength and elastic modulus of the RFAC gradually decreased, but its post-peak brittleness was significantly mitigated compared to NAC. The Poisson’s ratio of RFAC increased with the increase in the RGFA replacement ratio at the elastic stage and was smaller than that of NCA concrete. Both the existing stress–strain models developed for NAC and recycled aggregate concrete (RAC) were found not fit for the RFAC. Thus, a new stress–strain model that was applicable to RFAC was developed by modifying the classical existing model, and a good agreement between the model predictions and test data was reached.

## 1. Introduction

Being equipped with advantages such as lightweight, high strength and corrosion resistance, fiber-reinforced polymer (FRP) has gained extensive application in various industries, including construction, shipbuilding, wind power industry and aerospace industry [1,2,3,4,5,6]. In Europe, about 77,000 wind turbines were installed in 2018 alone, and the service life of the entire wind power project is only around 20 years, which will generate a large amount of FRP (original material of wind turbine blades) wastes at the end of the service life of wind power facility [7]. Besides, according to a report [8], the wind turbine will be a more reliable approach to achieve the goal of net-zero carbon dioxide emissions by 2050, which will further promote the quantity of FRP products used in the wind power industry. According to the glass FRP (GFRP) recycling economy study issued by Composites UK [9], the UK generates about 6200 tons of GFRP wastes and a potential 75,000 tons of GFRP scrap per year. In addition, the world market for continuous fiber-reinforced composite products reached USD 95 billion by 2020 [10]. Such a rapid increase in FRP applications will be accompanied by a large amount of waste generated during production, in-service and at the end of service life. Therefore, the disposal of the enormous quantity of FRP wastes has been a worldwide problem.

In the past, landfill or incineration was the usual treatment of FRP waste. However, it is expected that the landfill cost will increase significantly in the future [11] and that the awareness of the environmental impacts spreads widely [12], which will hinder waste disposal in this manner. Incineration is a less commonly used method for the treatment of thermosetting polymer composites (including FRP wastes) [12,13]. Due to the high calorific value of FRP materials [14], certain energy can be recovered from the heat generated during the combustion process. However, the cost of FRP incineration is higher than that of landfills, and the negative impact on the environment is much greater than that of landfills.

At present, mechanically recycling wastes GFRP and applying them into the concrete mixture as one of the ingredients is an eco-friendly and energy-saving approach [15,16,17]. In this regard, the large piece of FRP wastes is mechanically breaking up into smaller chunks (or fragments) to replace coarse aggregates or grinding into smaller powders as a replacement of fine aggregates. For recycling FRP as fine aggregates, some researchers found that the compressive strength of mortar or concrete decreases constantly with the increase of FRP replacement ratio [18,19,20,21,22,23]. Existing studies have shown that partially replacing fine aggregates with mechanically recycled FRP powder exerts no negative impact on the durability of Portland cement concrete and mortar. Tittarelli and Moriconi [18] studied the alkali-silicon reactivity of powdered GFRP, and the results did not reveal any potentially harmful reactions. However, replacing fine aggregate with recycled FRP powder resulted in worse workability [20,21]. When the volume of GFRP replaced sand reached 5–10%, the self-shrinkage will obviously increase [18].

**Figure 1:**
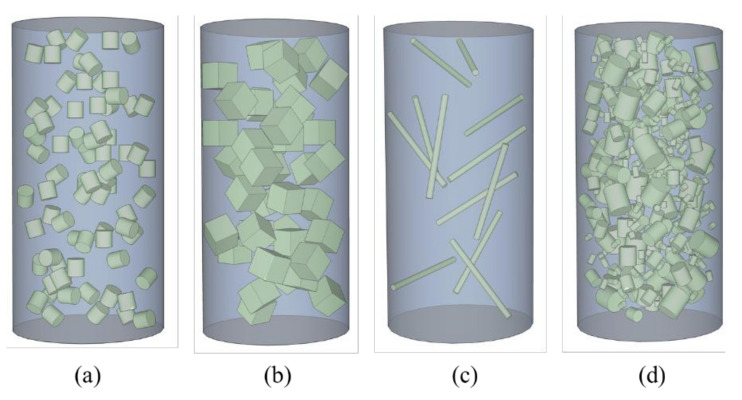
Recycled FRP aggregates concrete with different FRP aggregate shapes: (**a**,**b**) particles with single size; (**c**) needle shape; (**d**) particles with the gradation.

For recycling FRP into granular to replace the natural coarse aggregates (NCA) in concrete (Figure 1), the mechanical properties of this kind of concrete are gradually weakened with the increase of the replacement ratio, regardless of recycling FRP rebars or wind turbine blades. Yazdanbakhsh et al. [24,25] replaced NCA with discarded FRP bars that cut into granular form, which resulted in a varying reduction in compressive strength with the change of replacement ratio. Similar findings were obtained by Fox [26] and Alam et al. [27] who adopted composites waste from discarded wind turbine blades and waterslide casting. The reason can be due to the smooth surface of the FRP that weakened the interfacial bonding between the FRP surface and cement paste, which had been clarified by Yazdanbakhsh et al. [24,25].

Besides, there is some research related to making FRP rebar into “needles” to replace NCA in concrete. Yazdanbakhsh et al. [25] found a significant improvement in the tensile properties of concrete containing FRP needles (recycled from GFRP reinforcement with a diameter of 6 mm), while the compressive strength was still weakened. However, when Yazdanbakhsh et al. [28] replaced the FRP rebar with wind turbine blades to produce “needles” to replace NCA in concrete, no obvious degradation was found for the compressive strength. In contrast, a reduction in splitting tensile strength was found. Nie et al. [29] found that the surface conditions of recycled FRP needles showed a certain influence on the compressive and tensile strength of concrete. Therefore, the mechanical properties of concrete are found to be influenced by the resource, shape and interfacial conditions of recycled FRP aggregates (RFA) [24,25,26,27,28,29].

The above literature review indicates that the research on recycled FRP aggregates concrete (RFAC) is limited and that no relevant stress–strain model can be found in the open literature at present. Therefore, in this paper, waste GFRP rebar was recycled by cutting into particles to replace the NCA in concrete. Aggregate grading was considered when manually recycling GFRP bars. The effect of different GFRP volume replacement ratios on the performance of concrete was investigated, including failure mode, compressive strength, modulus of elasticity, lateral dilation and stress–strain response. The test results showed that the compressive strength and modulus of elasticity decreased with increasing RFA replacement ratio and that the failure mode of RFAC was different from that of natural aggregate concrete (NAC). Comparisons between test results and existing constitutive laws indicated that the stress–strain models originally proposed for NAC and recycled aggregate concrete (RAC, recycled aggregate here refers to aggregates recycled from construction and demolished wastes unless otherwise stated) cannot be used directly for RFAC. To better understand the behavior of RFAC under compression, a new and accurate stress–strain model is developed.

## 2. Experimental Program

### 2.1. Specimen Design

The waste GFRP bars were manually cut into short cylindrical (the diameter kept the same as the respective GFRP bar) particles with the length determined by considering aggregate grading, which was then used to replace NCA with different volume percentages to form the so-called RFAC. To investigate the effect of volumetric replacement ratio of GFRP aggregates on the axial compressive behavior of RFAC, this paper designed 15 standard cylinder specimens (150 mm in diameter and 300 mm in height) with the recycled GFRP aggregates (RGFA) replacement ratio of 0%, 30%, 50%, 70% and 100% considered, as summarized in Table 1. There were three replicate specimens for each replacement ratio. Each specimen was identified by a designation; for example, specimen FRP30 represents 30% (volume ratio) of NCA was replaced by RGFA aggregates. The mix proportion for each batch of the specimen is summarized in Table 1. This mix was designed under a water/cement ratio of 0.45, which corresponds to a cubic compressive strength of about 40 MPa. One day after casting, specimens were put into the standard curing room for 28 days, where the temperature and relative humidity was 20 ± 2 °C and 95%, respectively [30].

### 2.2. Material Properties

**Figure 2:**
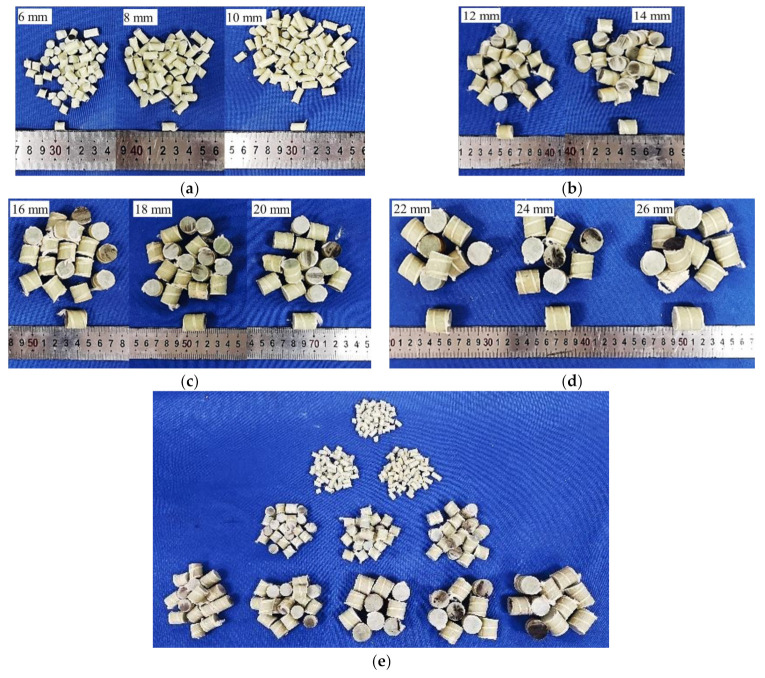
The cut scrap GFRP aggregates: (**a**) diameter of 6 mm; (**b**) diameter of 12 mm; (**c**) diameter of 16 mm; (**d**) diameter of 22 mm; (**e**) aggregates considering gradation.

**Figure 3:**
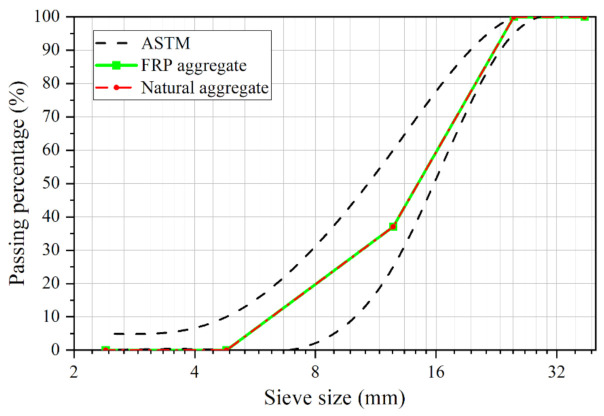
The grading of recycled GFRP aggregates and natural coarse aggregates.

**Figure 4:**
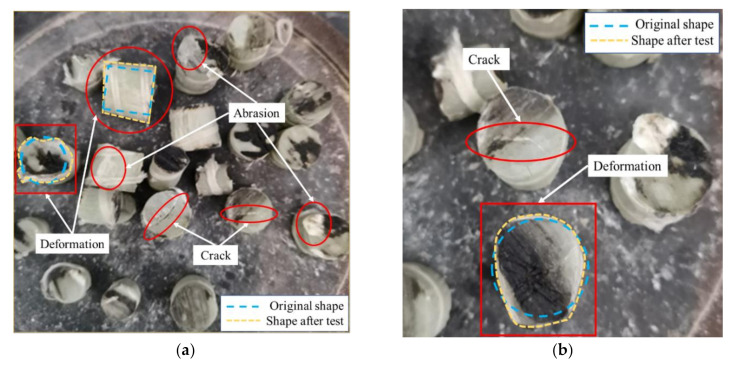
The GFRP aggregates after compression: (**a**) global view; (**b**) local perspective.

The materials used in the test were ordinary Portland cement (OPC) 42.5R, water, fine aggregates (river sand), NCAs (crushed stone) and RGFAs. The RGFAs used in the test were obtained by manually cutting the GFRP rebars with diamond saw blades, which included four kinds of diameters, i.e., 6 mm, 12 mm, 14 mm and 22 mm. The GFRP straight bars (no sand-coating) were provided by a local supplier (Shenzhen Haichuan New Material Co., LTD., Shenzhen, China). The mechanical properties of the GFRP bars are summarized in Table 2. As shown in Figure 2, the GFRP bars with a diameter of 6 mm were cut into cylinders with lengths of 6, 8 and 10 mm (Figure 2a); the ones with a diameter of 12 mm were cut into cylinders with lengths of 12 and 14 mm (Figure 2b); the ones with a diameter of 16 mm were cut into cylinders with lengths of 16, 18 and 20 mm (Figure 2c); and the ones with a diameter of 22 mm were cut into particles with lengths of 22, 24 and 26 mm (Figure 2d). Then, cylinder RGFAs of different sizes were mixed according to particle grading as per ASTM C33 [31], and the grading curve of RGFAs is shown in Figure 3. At the same time, the NCAs (with sizes ranging from 5 mm to 30 mm) used in this test shared the same aggregate gradation as that of RGFAs. In this way, the effect of different aggregate gradations on the strength of concrete after replacing NCAs with RGFAs can be minimized. On the one hand, the better continuity of RGFA gradation was considered. On the other hand, the length–diameter ratio of aggregates was controlled as low as possible (<1.6) to avoid negative effects caused by long and narrow GFRP aggregate particles. The properties of NCAs and RGFAs are summarized in Table 3. The crushing index of two kinds of aggregates was measured according to the specification JGJ 52-2006 [32]. The crushing results showed that the GFRP particles did not produce crushed fine particles as well as powders after the test, whereas, only cracks or deformation were generated on the particles, as shown in Figure 4a,b.

### 2.3. Test Setup

**Figure 5:**
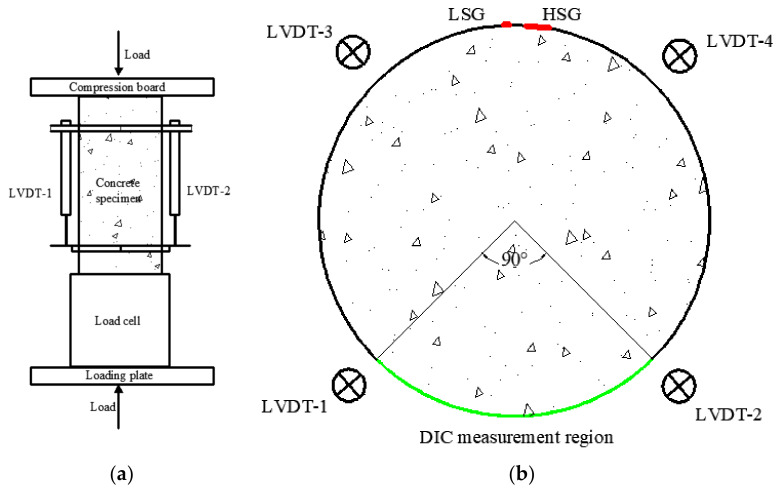
Sketch of test setup: (**a**) front view; (**b**) sectional view.

**Figure 6:**
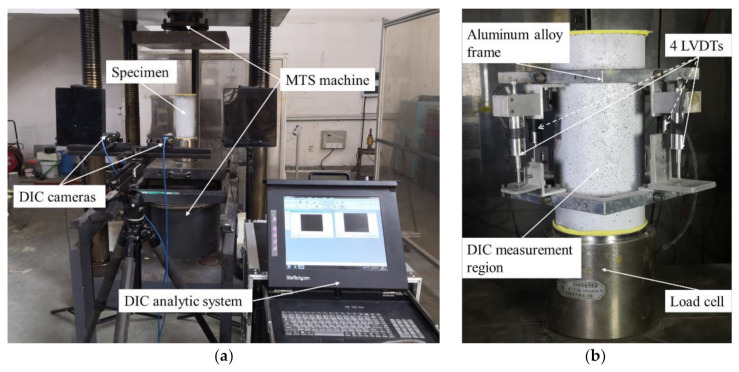
Pictures of uniaxial compression test: (**a**) experimental system; (**b**) instrumentations.

The uniaxial compression test was carried out using a 3000KN MTS, and a displacement control loading mode (0.3 mm/min) was adopted. Four longitudinal linear variable differential transformers (LVDTs) were fixed on the body of each specimen to monitor the longitudinal displacements, as shown in Figure 5 and Figure 6b. The positions of four LVDTs were evenly distributed along the perimeter of a specimen, and the gauge length was 185 mm, as illustrated in Figure 5. The digital imagine collection (DIC) system was used to record the strain fields of all specimens, and the DIC covered area is shown in Figure 5b and Figure 6. For each specimen, a longitudinal strain gauge (LSG) and a hooped strain gauge (HSG) were installed in the middle height (the back of the DIC specialized surface) to measure the strain of concrete, as depicted in Figure 5b. In this way, multiple strain measurements were obtained to evaluate the accuracy of the data and reduce the possible errors. Before the formal test, all cylinders were leveled with plaster on the upper and lower surfaces to obtain horizontally smooth loading and supporting surfaces.

## 3. Results and Discussions

### 3.1. Failure Modes

**Figure 7:**
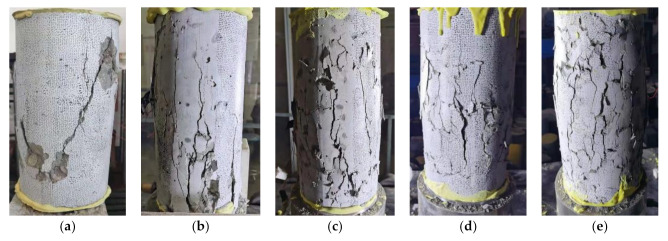
Failure modes: (**a**) FRP0; (**b**) FRP30; (**c**) FRP50; (**d**) FRP70; (**e**) FRP100.

**Figure 8:**
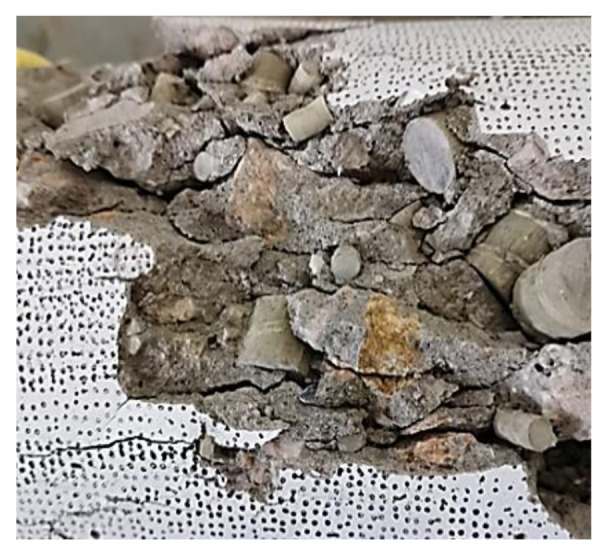
The inner cracks patterns of RFAC specimens FRP70.

Figure 7 shows the failure modes of specimens with different replacement ratios of RGFAs. It can be seen from the figure that there was a main diagonal crack for NCA concrete specimens (FRP0 group). However, for specimens containing RGFAs, two different phenomena were observed. First, many more cracks with no obvious inclination rather than one major diagonal crack were formed for RFAC specimens, which had not been depicted in other research work [24,25,26,27]. Second, a much more significant lateral dilation was observed for all RFAC specimens, and the extent of lateral dilation increased with increasing replacement ratios of RGFAs. The relatively regular shape and smooth surface of GFRP particles resulted in a weak bond between the particles and the surrounding cement matrix [24], which triggered micro-cracks to develop between the GFRP particles and the matrix during loading (Figure 8). Eventually, RFAC specimens ended in a significantly different crack profile at failure when compared to NCA concrete specimens.

### 3.2. Stress–Strain Curves

**Figure 9:**
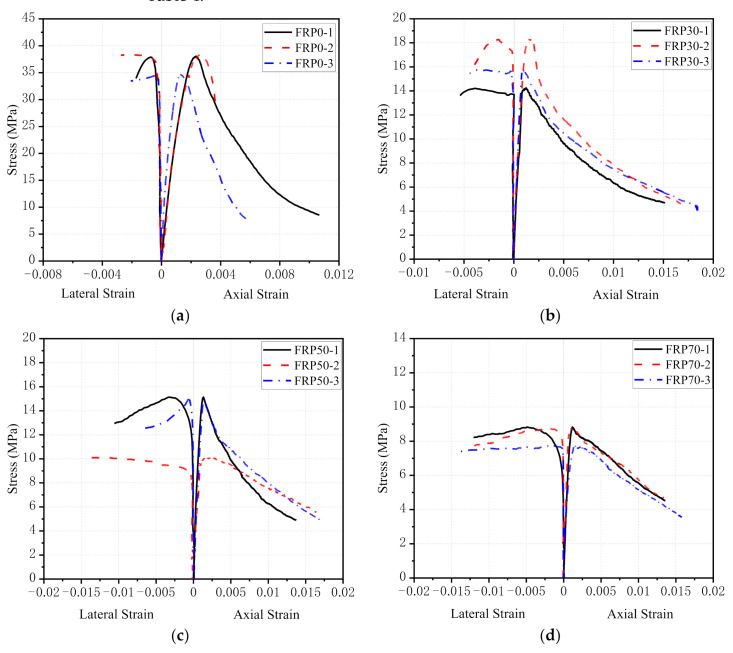

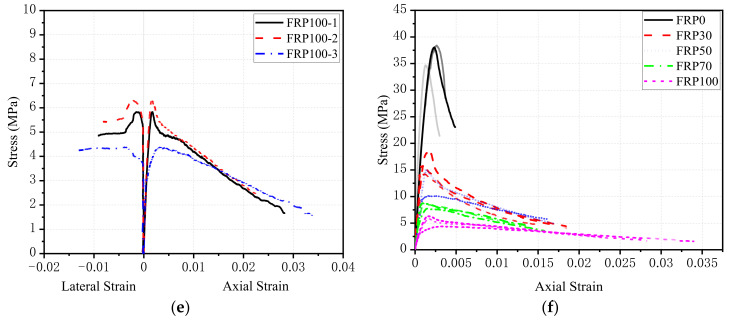
Compressive stress–strain curves of specimens under different RGFA replacement ratios: (**a**) 0%; (**b**) 30%; (**c**) 50%; (**d**) 70%; (**e**) 100%; (**f**) 0–100%.

Figure 9 depicts the stress–strain responses for RFAC specimens. The stress was calculated according to the data collected by the force sensor. The strain was obtained by taking the averaged data of four LVDTs, which were compared with the data captured by LGS and DIC. Key points on the axial stress and axial strain curve, including peak stress, peak strain (at peak stress) and elastic modulus of RFAC specimens, are summarized in Table 4.

As shown in Figure 9, for axial stress and axial strain curves, general findings are that the modulus of elasticity and compressive strength decreased with the increasing replacement ratio of RGFA. Besides, the post-peak softening response was significantly mitigated by increasing the RGFA replacement ratio, indicating a good toughness of RFAC specimens. Figure 9f compares the axial stress and axial strain curves of all RFAC specimens. For other kinds of recycled concrete, e.g., shale ceramsite lightweight aggregate concrete [33], recycled brick aggregate concrete and recycled rubber concrete [34,35], similar trends that the extent of post-peak softening was improved as the replacement ratio increased. However, the research results of RAC from the demolition of concrete structures resulted in opposite conclusions. Some found that brittleness increased with the increase of the replacement ratio [36,37], whereas others showed that ductile failure was more likely to occur as the replacement ratio increased [38]. Figure 9a–e depicts the axial stress–lateral strain responses for RFAC specimens. It can also be seen that the RFAC specimens experienced larger strain than the NCA concrete specimen at the same magnitude of stress drop within the post-peak softening range of the stress–strain curve. Thus, RFAC became more susceptible to lateral expansion. The reason for this phenomenon will be given subsequently.

**Figure 10:**
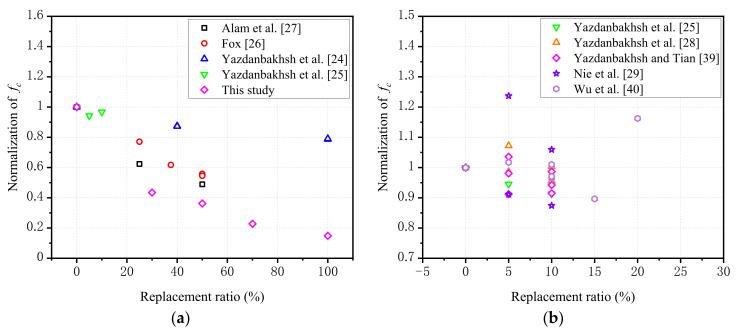
Compressive strength vs. FRP scraps replacement ratio (normalization of *f_c_* means the strength of FRP-concrete divided by the respective control specimen): (**a**) FRP particles; (**b**) FRP needles [24,25,26,27,28,29,39,40].

Table 4 showed that the compressive strength decreased with the increase of the replacement ratio of RGFA. When the replacement ratio was 30%, the compressive strength decreased by 56.52%, while when the RGFAs completely replaced the NCAs, the compressive strength of the RFAC specimen was only about one-seventh of the NCA specimen. Other studies showed that the compressive strength decreased by 45.4% when 50% of NCA was replaced by a single-size GFRP aggregate recycled from discarded wind turbine blades [26]. In addition, Alam et al. [27] found that the compressive strength of concrete with square FRP aggregate was reduced by more than 50% when the replacement ratio reached 50%. However, some studies indicated that the compressive strength of concrete with recycled GFRP particles decreased by only about 21% when the replacement ratio was 100% [24], and by 6% to 3% when the replacement ratios were within the range from 5% to 10% [25]. In general, the changing trend of compressive strength is consistent with previous studies, which can be found in Figure 10a. The FRP aggregates in references [24,25] were composed of five particle sizes according to the gradation composition of ASTM C33, Size No. 56 [31]. As summarized in Table 4, the elastic modulus of RFAC decreased gradually with the increase of the replacement ratio of RGFA, and the peak strain was also lower than that of the NCA concrete. Within the range of replacement ratio from 0% to 50%, the peak strain decreased first and then increased with the increase of the RGFA replacement ratio. When the replacement ratio over 50%, it also displayed the same changing pattern.

Based on the above comparisons and discussions, three reasons can be found for the strength reduction of RFAC. First, the surface of the RGFA mentioned above was smoother, and the shape was more regular than that of the NCAs. As a result, the interfacial transition zone (ITZ) between the RGFA and the cement paste was much weaker, resulting in a significant decline in its strength. Other researchers also made similar remarks [24,26]. Second, the RGFA may slowly absorb water during the 28 days of curing, so that the hydration degree of cement around the RGFA was lower, thus forming a weak ITZ [24]. Third, even though this paper tried to include more sizes to make the RGFA reach a more continuous gradation, the actual gradation of RGFA was still worse than the NCAs. As shown in Table 5, notably, the compressive strength degradation rate must be related to the properties of recycled FRP materials used in RFAC. Different studies resulted in significant differences in the specific reduction of compressive strength at the same FRP replacement ratio when the different materials were used, as depicted in Figure 10a. The normalized $f_{c}$ refers to the ratio of the compressive strength of RFAC to that of the corresponding control specimen. In Figure 10b, the FRP aggregate is a “needle” shape, whose length is much longer than its diameter, approaching 100 mm.

### 3.3. Axial Strain–Lateral Strain Relationship

**Figure 11:**
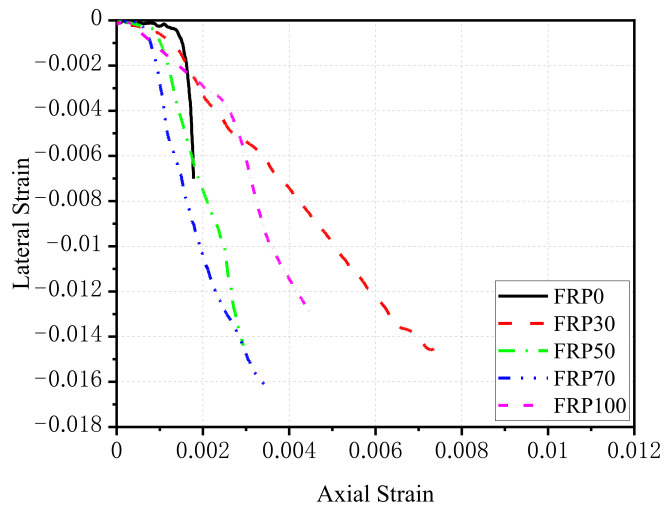
Axial strain–lateral strain response of RFAC specimens.

Figure 11 shows the relationship between axial strain and lateral strain for all specimens. For NAC specimens (FRP0 group), when the stress of concrete was low, the curve developed almost linearly. The slope of the curve increased sharply when the axial strain reached about 60–80% of the peak strain, which was caused by the rapid development of micro-cracks in concrete. For RFAC, the lateral deformation of the FRP30 group was larger than that of NCA concrete when $\varepsilon_{c}^{\prime }<0.002$. At larger $\varepsilon_{c}^{\prime }$, the lateral strain of NCA concrete increased rapidly until failure occurred, whereas the lateral strain of the FRP30 group increased much slower but reached much larger values than that of NCA concrete at failure. When the replacement ratio of RGFA ranged from 30% to 70%, the lateral strain increased with increasing replacement ratio under the same axial strain, which reflected that the more RGFA content resulted in a greater lateral expansion of concrete, as shown in Figure 7. When $\varepsilon_{c}^{\prime }<0.003$, the axial and lateral strain curve of the FRP100 was similar to that of FRP30. With the further increase of $\varepsilon_{c}^{\prime }$, the lateral strain of FRP100 was much larger than that of FRP30 at the same axial strain. However, the lateral strain of FRP100 was smaller than that of FRP70 and FRP50 at the same axial strain when the axial strain was larger than a certain value.

**Figure 12:**
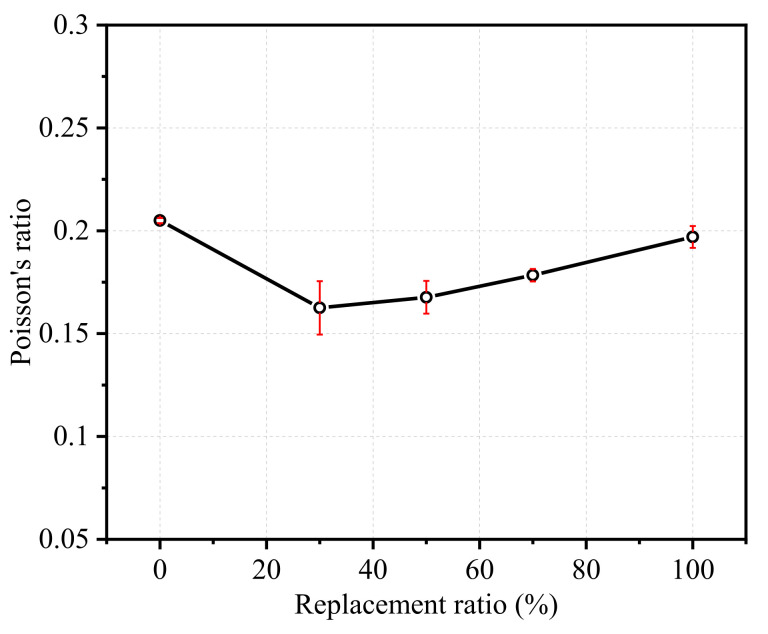
The Poisson’s ratio of RFAC specimens.

Figure 12 compares the Poisson’s ratios of all specimens. The ratio of transverse strain to axial strain is Poisson’s ratio, which was originally meant to be an elastic constant and a characteristic of isotropic, homogeneous and elastic materials [41]. The Poisson’s ratio of the NCA specimen was found to be around 0.21 (Figure 12) and the Poisson’s ratio of RFAC specimens was ranged from 0.14 to 0.20, which were within the variation range of different kinds of concrete, e.g., NAC concrete, RAC and alkali-activated concrete [42,43,44,45]. The Poisson’s ratio of RFAC specimens was dependent on the content RGFA. The Poisson’s ratio of FRP30 was the smallest. Then, when the replacement ratio increased, the Poisson’s ratio also gradually increased, and it finally reached a value that was close to that of NCA concrete when the RGFA replacement ratio was 100%.

## 4. Development of a Stress–Strain Model for RFAC

### 4.1. The Need for a New Stress–Strain Model of RFAC

The stress–strain relationship of concrete under compression is an important basis to quantitatively evaluate the mechanical properties. For both NCA concrete [46,47,48,49,50,51,52] and normal RAC [38,52,53,54,55], various models are available to predict the stress–strain relationships at present. However, for RFAC, to the best knowledge of the authors, no stress–strain models can be found in the open literature so far. In this section, some typical stress–strain models that were originally developed for NCA concrete and normal RAC were reviewed, discussed and evaluated using the stress–strain curves of RFAC specimens. Then, a new model that can be used to predict the stress–strain response of RFAC was developed.

#### 4.1.1. Stress–Strain Models for Normal Concrete

In this section, four representative stress–strain models of NCA concrete were selected to compare with the test curves (only one test curve in each group was used), the expressions of which are summarized in Table 6. The expressions of stress–strain models proposed by Chu et al. [47] and Yi et al. [50] are similar, and the main parameters that affect the curve shape are peak strain $\varepsilon_{c}^{\prime }$ and a material parameter *β*. For the model proposed by Chu et al. [47], the strength $f_{c}^{\prime }$, peak strain $\varepsilon_{c}^{\prime }$ and elastic modulus $E_{c}$ are taken into account in the expression of *β*, while in the model of Yi et al. [50], the curing time *t*, elastic modulus $E_{c}$ and secant modulus $E_{0}$ are considered. The Hognestad’s model [49] is divided into ascending and descending portions, whereas model in EU code [48] adopts a continuous equation.

**Figure 13:**
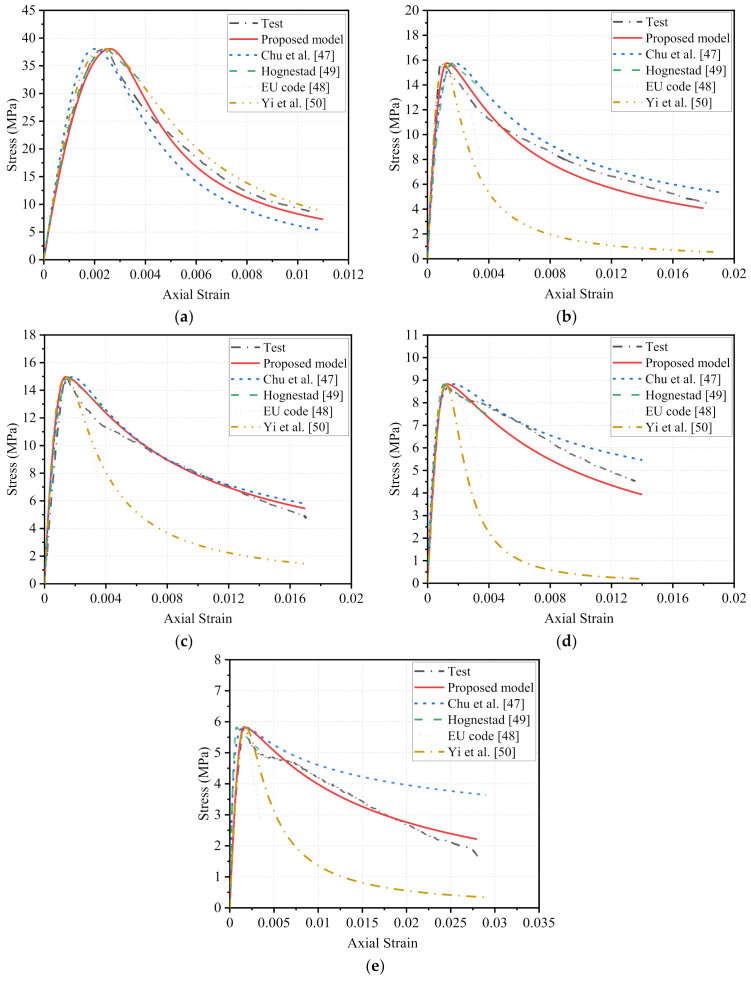
Performance of proposed model and existing models developed for normal concrete: (**a**) *r* = 0, (**b**) *r* = 30%, (**c**) *r* = 50%, (**d**) *r* = 70%, (**e**) *r* = 100%.

Figure 13 compares the stress–strain curves predicted by the above-stated four models and obtained in the tests. For the ascending part of the curves, all the models can give a relatively close prediction regardless of NCA concrete or RFAC. But the accuracy of some models was slightly weakened as the content of the RGFA changed. For the post-peak softening part of the curve, the accurate predictions for the specimen in the FRP0 group were also reached. For RFAC, the predictions of the models developed by Chu et al. [47] and Hognestad [49] were relatively close to the test data, and the other two models were relatively lower than the test curve, and the deviation was larger when the replacement ratio of RGFA was high. This is reasonable that all four models are developed for NCA concrete rather than RFAC.

#### 4.1.2. Stress–Strain Models for Recycled Concrete

**Figure 14:**
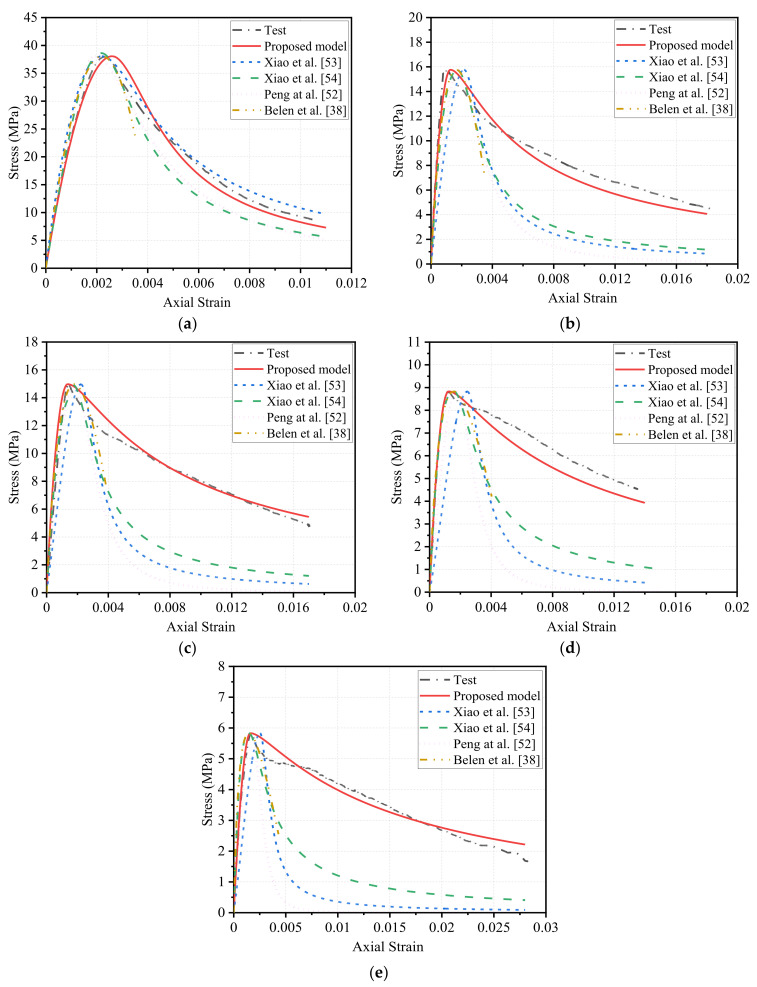
Performance of proposed model and existing models developed for recycled concrete: (**a**) *r* = 0, (**b**) *r* = 30%, (**c**) *r* = 50%, (**d**) *r* = 70%, (**e**) *r* = 100%.

In terms of RAC, four existing stress–strain models were also selected for comparison, and the specific expressions are summarized in Table 7. The model of Xiao et al. [53] was proposed based on the framework developed by Guo and Zhang [51], which obtained the expressions of shape parameters *a* and *b* by considering the recycled aggregate replacement ratio *r*. In addition, Xiao et al. [54] adjusted the expressions of peak strain $\varepsilon_{c}^{\prime }$, elastic modulus $E_{c}$ and shape parameter $\alpha_{c}$ to make the model more suitable for recycled concrete. The stress–strain model of Peng et al. [52] was modified from the model of Chu et al. [47] by considering different material parameters β in ascending and descending stages. Based on the stress–strain model of the EU Code [48], Belen et al. [38] introduced the conversion coefficient of recycled aggregate to adjust secant modulus $E_{cm}$, peak strain $\varepsilon_{c}^{\prime }$ and ultimate strain $\varepsilon_{cu}$. The comparison between stress–strain curves predicted by the above-stated models and measured in tests is shown in Figure 14. As expected, for specimen in FRP0 group, the predictions were highly consistent with the test data. However, for RFAC, the discrepancies between the models’ predictions and test results were relatively large. The disagreements between the existing models and test data are reasonable since they were proposed originally for RAC rather than RFAC. Thus, the development of a new model that can be used to predict the stress–strain response of RFAC is still needed.

### 4.2. Proposed Model for RFAC

Based on the analytical constitutive law proposed by Guo and Zhang [51], which was originally developed for NCA concrete, a new stress–strain model that is able to predict the uniaxial compressive stress–strain response of RFAC was developed in this section. The model was expressed by ascending and descending portions separately, the expression of which are described in Equations (1) and (2):

(1) $$\frac{f_{c}}{f_{c}^{\prime }}=a\frac{\varepsilon_{c}}{\varepsilon_{c}^{\prime }}+\left(3-2a\right){\left(\frac{\varepsilon_{c}}{\varepsilon_{c}^{\prime }}\right)}^{2}+\left(a-2\right){\left(\frac{\varepsilon_{c}}{\varepsilon_{c}^{\prime }}\right)}^{3}\;\;0\;\leq \;\frac{\varepsilon_{c}}{\varepsilon_{c}^{\prime }}\;\leq \;1$$

(2) $$\frac{f_{c}}{f_{c}^{\prime }}=\frac{\varepsilon_{c}/\varepsilon_{c}^{\prime }}{b{\left(\varepsilon_{c}/\varepsilon_{c}^{\prime }-1\right)}^{2}{+\varepsilon }_{c}/\varepsilon_{c}^{\prime }}\;\frac{\varepsilon_{c}}{\varepsilon_{c}^{\prime }}\;\geq \;1$$

(3) $$\varepsilon_{c}^{\prime }=\frac{2f_{c}^{\prime }}{E_{c}}$$

(4) $$E_{c}{=4730k}_{e}\cdot \sqrt{f_{c}^{\prime }}$$

(5) $$k_{e}=1+1.630\sqrt{r}-2.090r$$

**Figure 15:**
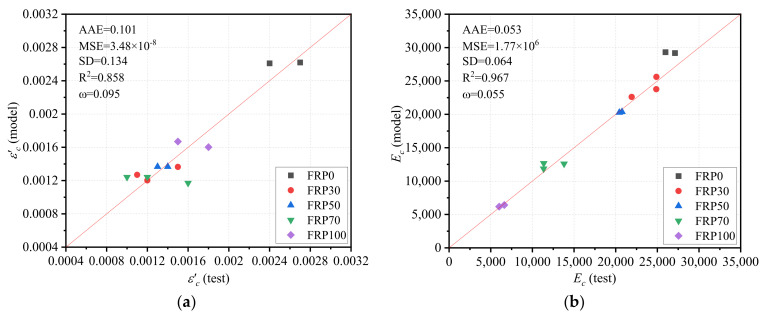
Performance of the proposed model: (**a**) strain corresponding to peak stress $\varepsilon_{c}^{\prime }$; (**b**) elastic modulus $E_{c}$.

In the new stress–strain model, the peak strain $\varepsilon_{c}^{\prime }$ adopted the expression proposed by Hognestad [49], as expressed in Equation (3). Equation (4) gives the expression of elastic modulus $E_{c}$ occurred in Equation (3), which was modified based on the model for NAC concrete specified in ACI 318 standard [56], where a coefficient $k_{e}$ that is related to the RGFA replacement ratio *r* was introduced, as expressed in Equation (5). A comparison between the predictions of Equation (3) and the experimentally measured values is shown in Figure 15a, where the AAE, MSE, and SD values are all small, indicating the accuracy of the modified models. Figure 15b shows the performance of the proposed model for $E_{c}$, where the AAE and $R^{2}$ were found to be less than 0.1 and greater than 0.9, respectively, indicating the good performance of the proposed model for the elastic modulus of RFAC.

In addition, the coefficients *a* and *b* in Equations (1) and (2) are specific parameters for a given strength level of the concrete. The coefficient *a* is the shape parameter that controls the ascent stage of the curve, which is equal to the initial elastic modulus divided by the cutline modulus at the peak point ($a=\frac{E_{0}}{E_{1}}$) [52]. The coefficient *b* dominates the shape of the descending stage of the stress–strain curve, and a larger value results in a steeper curve, and vice versa. The coefficients *a* and *b* were modified with the RGFA replacement ratio *r* included. By regressing analysis against test data, the expressions for *a* and *b* were derived as follows:

(6) $$a=1.927+0.489r+1.230r^{2}-1.333r^{3}$$

(7) $$b=0.129{(r+0.011)}^{-0.571}$$

Figure 13 or Figure 14 compares the stress–strain curves predicted by the proposed model and measured in tests. It can be found that key features of the stress–strain behavior can be captured by the new model. Particularly, good agreement between model prediction and test data was observed for the ascending part of the stress–strain curve of RFAC under varying replacement ratios of RGFA. For the post-peak softening stage, when the RGFA replacement ratio ranged from 50% to 100%, the stress in tests suddenly and sharply decreased shortly after the peak stress, thereafter the stress decline rate tended to be moderate when the axial strain reached certain values, as shown in Figure 14c–e. This unsmooth portion of the curve was not well captured by the new model described in Equations (1) and (2). Although the above-stated local differences existed between the model predictions and test results, the post-peak softening responses calculated as per the developed new model generally matched the experimental data.

## 5. Conclusions

In this paper, waste GFRP bars were cut into 12 kinds of granular shapes (with aggregate gradation considered) to replace NCA to form the so-called RFAC. The main test variable of standard RFAC cylinders was the volume replacement ratios of RGFA, i.e., 0%, 30%, 50%, 70% and 100%. Uniaxial compressive tests were conducted to evaluate the effect of RGFA replacement ratios on the mechanical properties of RFAC, including compressive strength, peak strain, modulus of elasticity and stress–strain response. Based on the existing constitutive law, a new stress–strain model was modified and developed for RFAC. The following conclusions can be drawn:

1. The failure mode of RFAC was different from that of NCA concrete. Unlike NCA concrete failed by an obvious main diagonal crack, the cracks of RFAC were found to be distributed without an obvious main diagonal crack. This phenomenon was more significant as the RGFA replacement ratio increased.
2. The compressive strength $f_{c}^{\prime }$ of RFAC decreased non-linearly with the increase of the RFA replacement ratio, which was also true for the elastic modulus. The strain corresponding to the peak stress decreased compared with that of NCA concrete.
3. Under the same axial stress, RFAC tended to expand more easily (reflected by larger lateral strain), especially in the post-peak softening stage. The Poisson’s ratio of RFAC at the elastic stage was slightly smaller than that of NCA concrete and tended to increase with the increase of the RGFA replacement ratio, but the overall change range was small, ranging from 0.14 to 0.20.
4. The shape of the stress–strain curve of RFAC was different from that of NCA concrete, especially in the post-peak softening range. This can be due to the following changes of RFAC, including the weaker interfacial bond between GFRP aggregates and cement matrix, larger lateral expansion under the same axial stress and different cracking pattern.
5. Comparisons between the test results and existing stress–strain models developed for NCA concrete as well as RAC indicated that the existing models are not suitable for predicting the stress–strain relationship of RFAC. Thus, based on the existing classical models, by considering the RGFA replacement ratio, a set of new models were proposed to predict the modulus of elasticity, peak strain and stress–strain model of RFAC, and good agreements were found between the models’ predictions and test results.

The conclusions drawn from the current study are based on the test results, discussions and theoretical modeling presented above. Notably, due to the fact that only limited ranges of parameter variation were considered in this work and that no other existing stress–strain curves can be found in the open literature, further studies are needed to substantiate and further improve the findings presented in the current work. Future research work can be carried out in improving the interfacial properties between FRP aggregate and mortar, and developing methods to enhance the compressive behavior of RFAC can also be an interesting area.

## Figures and Tables

**Table 1:** Specimen details.

Spec. ID	Recycled GFRP Replacement Ratio (vol.%)	Mix Proportions (kg/m^3^)					
		W/C	Water	OPC	Natural Aggregate	Recycled GFRP Aggregate	Sand
FRP0	0	0.45	190	422	950	0	683
FRP30	30	0.45	190	422	666	225	683
FRP50	50	0.45	190	422	475	375	683
FRP70	70	0.45	190	422	285	526	683
FRP100	100	0.45	190	422	0	751	683

**Table 2:** Properties of GFRP rebars.

Indicators	Value (Type)
Tensile strength (MPa)	920–1230
Tensile modulus of elasticity (GPa)	53
Density (kg/m^3^)	1933–2070
Fiber content by weight (%)	78
Fiber type	*E* glass fiber
Matrix type	Unsaturation resin

**Table 3:** Properties of two kinds of aggregates.

Type	Bulk Density (kg/m^3^)	Crushing Value (wt.%)	Cylinder Compressive Strength (MPa)
Natural aggregate	1513.67	9.55	19.43
Recycled GFRP aggregate	1195.67	\	36.35

**Table 4:** The compressive test result of unconfined specimens.

Spec. ID	*r* (%)	*f’_c_* (MPa)	Avg. (MPa)	Change ^a^ (%)	*E_c_* (GPa)	Avg. (GPa)	Change ^b^ (%)	*ε′_c_* (%)	Avg. (%)	Change ^c^ (%)
FRP0-1	0	38.06	37.04	/	27.11	26.54	/	0.24	0.26	/
FRP0-2	0	38.40			25.97			0.27		
FRP0-3	0	34.67			52.91			0.13		
FRP30-1	30	14.26	16.11	−56.52	21.92	23.89	−9.97	0.12	0.13	−50.00
FRP30-2	30	18.30			24.89			0.15		
FRP30-3	30	15.76			24.87			0.11		
FRP50-1	50	15.13	13.42	−63.76	20.76	18.77	−29.28	0.13	0.14	−46.15
FRP50-2	50	10.17			15.10			0.25		
FRP50-3	50	14.97			20.44			0.14		
FRP70-1	70	8.83	8.43	−77.23	11.36	12.17	−54.14	0.12	0.13	−50.00
FRP70-2	70	8.74			13.80			0.10		
FRP70-3	70	7.73			11.35			0.16		
FRP100-1	100	5.83	5.51	−85.13	6.01	7.48	−71.82	0.18	0.17	−34.62
FRP100-2	100	6.31			6.63			0.15		
FRP100-3	100	4.39			9.80			0.34		

Note: ^a^ Change = (*f’_c_* of RFAC − *f’_c_* of NAC)/*f’_c_* of NAC; ^b^ Change = (*E_c_* of RFAC − *E_c_* of NAC)/*E_c_* of NAC; ^c^ Change = (*ε’_c_* of RFAC − *ε’_c_* of NAC)/*ε’_c_* of NAC.

**Table 5:** Different test results in the present study on FRP aggregate replacing natural coarse aggregate.

Test	Replacement (%)	Change ^a^ (%)	Material	Gradation of FRP Aggregate
This study	0	/	GFRP rebars (*E* glass fibers and unsaturation resin)	ASTM C33 No.57 (Granular FRP aggregate size: 6 mm, 8 mm, 10 mm, 12 mm, 14 mm, 16 mm, 18 mm, 20 mm, 22 mm, 24 mm, 26 mm)
	30	−56.52		
	50	−63.76		
	70	−77.23		
	100	−85.13		
Alam et al. [27]	0	/	The excess from the casting of waterslides	CSA A23. (square FRP aggregate size: 10 mm–30 mm)
	25	−37.64		
	50	−51.09		
Fox [26]	0	/	Wind turbine blades	
	25	−22.96		Cubic FRP size: 25 mm
	37.5	−38.27		
	50	−45.43		
	50	−44.94		Cubic FRP size: 13 mm
	50	−45.43		Cubic FRP size: 13 mm (25%) + 25 mm (25%)
	50	−44.2		Cubic FRP size: 25 mm
Yazdanbakhsh et al. [24]	0	/	GFRP rebars (ECR glass fibers and vinyl Ester resin)	ASTM C33 No. 56 (graular FRP aggregate size: 6 mm, 10 mm, 13 mm, 16 mm, 19 mm, 25 mm)
	40	−12.53		
	100	−21.33		
	0	/		
	40	−12.74		
	100	−20.95		
Yazdanbakhsh et al. [25]	0	/	GFRP rebars (ECR glass fibers and vinyl Ester resin)	ASTM C33 No. 56 (graular FRP aggregate size: 6 mm, 10 mm, 13 mm, 16 mm)
	5	−5.72		
	10	−3.23		

Note: ^a^ Change = (*f’_c_* of RFAC − *f’_c_* of NAC)/*f’_c_* of NAC.

**Table 6:** Four representative expressions of normal concrete models.

Model	Expression	Other Parameters
Chu et al. [47]	$\frac{f_{c}}{f_{c}^{\prime }}=\frac{\beta (\frac{\varepsilon_{c}}{\varepsilon_{c}^{\prime }})}{\beta -1+(\frac{\varepsilon_{c}}{\varepsilon_{c}^{\prime }}{)}^{\beta }}$	$\beta =\frac{1}{1-\frac{f_{c}^{\prime }}{\varepsilon_{c}^{\prime }E_{c}}}$ $E_{c}=\frac{f_{c}^{\prime }}{\varepsilon_{c}^{\prime }}(\frac{24.82}{f_{c}^{\prime }}+0.92)$ $\varepsilon_{c}^{\prime }=(1680+7.1f_{c}^{\prime }{)\times 10}^{-6}$
Hognestad [49]	$\frac{f_{c}}{f_{c}^{\prime }}=\left\{\begin{array}{c} \left[\frac{{2\varepsilon }_{c}}{\varepsilon_{c}^{\prime }}-{\left(\frac{\varepsilon_{c}}{\varepsilon_{c}^{\prime }}\right)}^{2}\right]{,\;\varepsilon }_{c}{<\varepsilon }_{c}^{\prime }\\ \left[1-0.15(\frac{\varepsilon_{c}-{\;\varepsilon }_{c}^{\prime }}{\varepsilon_{cu}-{\;\varepsilon }_{c}^{\prime }})\right]{,\;\varepsilon }_{c}^{\prime }\leq \varepsilon_{c}\leq \varepsilon_{cu} \end{array}\right.$	$\varepsilon_{c}^{\prime }=\frac{2f_{c}^{\prime }}{E_{c}}$ $E_{c}=12411+460f_{c}^{\prime }$ $\varepsilon_{cu}=0.0038$
EU code [48]	$\frac{f_{c}}{f_{c}^{\prime }}=\frac{k\left(\varepsilon_{c}/\varepsilon_{c}^{\prime }\right)-{\;(\varepsilon }_{c}/\varepsilon_{c}^{\prime }{)}^{2}}{1+\left(k-2\right)\left(\varepsilon_{c}/\varepsilon_{c}^{\prime }\right)}$	$k=1.05E_{cm}\varepsilon_{c}^{\prime }/f_{c}^{\prime }$ $E_{cm}=22(\frac{f_{c}^{\prime }}{10}{)}^{0.3}$ $\varepsilon_{c\;}^{\prime }=0.7{f_{c}^{\prime }}^{0.31}$ $\varepsilon_{cu}=0.0035$
Yi et al. [50]	$\frac{f_{c}}{f_{c}^{\prime }}=\frac{\beta_{m}(\frac{\varepsilon_{c}}{\varepsilon_{c}^{\prime }})}{\beta_{m}-1+(\frac{\varepsilon_{c}}{\varepsilon_{c}^{\prime }}{)}^{\beta_{m}}}$	$\beta_{m}{=\beta }_{m,a}={\left[1.02-1.17(\frac{E_{0}}{E_{c}})\right]}^{-0.74}\;\varepsilon_{c}\leq \varepsilon_{c}^{\prime }$ $\beta_{m}{=\beta }_{m,d}{=\beta }_{m,a}+\left(a+bt\right)\;\varepsilon_{c}\geq \varepsilon_{c}^{\prime }$ $a=\;{\left(12.4-1.66{\times 10}^{-2}\cdot f_{28}\right)}^{-0.46}$ $b=0.83exp(-\frac{911}{f_{28}})$

**Table 7:** Four representative expressions of recycled concrete models.

Model	Expression	Other Parameters
Xiao et al. [53]	$\frac{f_{c}}{f_{c}^{\prime }}=\left\{\begin{array}{c} a\frac{\varepsilon_{c}}{\varepsilon_{c}^{\prime }}+\left(3-2a\right){\left(\frac{\varepsilon_{c}}{\varepsilon_{c}^{\prime }}\right)}^{2}+\left(a-2\right){\left(\frac{\varepsilon_{c}}{\varepsilon_{c}^{\prime }}\right)}^{3},\;0\leq \frac{\varepsilon_{c}}{\varepsilon_{c}^{\prime }}\leq 1\\ \frac{\varepsilon_{c}/\varepsilon_{c}^{\prime }}{{b(\varepsilon }_{c}/\varepsilon_{c}^{\prime }-{1)}^{2}+\;\varepsilon_{c}/\varepsilon_{c}^{\prime }},\;\frac{\varepsilon_{c}}{\varepsilon_{c}^{\prime }}\geq 1 \end{array}\right.\;$	$a=2.2(0.748r^{2}-1.231r+0.975)$ b=0.8(7.6483r+1.142)
Xiao et al. [54]	$f_{c}=(1-d_{c}{)E}_{c}\varepsilon_{c}$ $d_{c}=\left\{\begin{array}{c} 1-\frac{\rho_{c}m_{1}}{m_{1}-{1+(\varepsilon }_{c}/\varepsilon_{c}^{\prime }{)}^{m_{1}}}\;0\leq \frac{\varepsilon_{c}}{\varepsilon_{c}^{\prime }}\leq 1\;\\ 1-\frac{\rho_{c}}{\alpha_{c}{{(\varepsilon }_{c}/\varepsilon_{c}^{\prime })}^{2}{+(\varepsilon }_{c}/\varepsilon_{c}^{\prime })}\;0\leq \frac{\varepsilon_{c}}{\varepsilon_{c}^{\prime }}>1 \end{array}\right.$	$\rho_{c}=\frac{f_{c}^{\prime }}{E_{c}\varepsilon_{c}^{\prime }}\;m_{1}=\frac{E_{c}\varepsilon_{c}^{\prime }}{E_{c}\varepsilon_{c}^{\prime }\;-\;f_{c}^{\prime }}$ $\varepsilon_{c}^{\prime }=m\cdot \sqrt{f_{c}^{\prime }}+n\;$ $E_{c}=\frac{10}{p+q/f_{c}^{\prime }}$ $\alpha_{c}=\mu \cdot {f_{c}^{\prime }}^{0.785}-\nu$
Peng et al. [52]	$\frac{f_{c}}{f_{c}^{\prime }}=\frac{\beta (\frac{\varepsilon_{c}}{\varepsilon_{c}^{\prime }})}{\beta -1+(\frac{\varepsilon_{c}}{\varepsilon_{c}^{\prime }}{)}^{\beta }}$	$\beta \;=\left\{\begin{array}{c} 4.23{\times 10}^{-6}\cdot exp\left(\frac{15.74E_{0}}{E_{c}}\right)\\ +3.38\\ \varepsilon_{c}\leq \varepsilon_{c}^{\prime }\;\\ 0.8\beta_{a}-0.803+0.0204f_{max0}\\ +0.7933exp\left(\frac{43.15}{f_{max0}}\right)r\\ \varepsilon_{c}\geq \varepsilon_{c}^{\prime } \end{array}\right.$
Belen et al. [38]	$\frac{f_{c}}{f_{c}^{\prime }}=\frac{k\left(\varepsilon_{c}/\varepsilon_{c}^{\prime }\right)-{\left(\varepsilon_{c}/\varepsilon_{c}^{\prime }\right)}^{2}}{1+\left(k-2\right)\left(\varepsilon_{c}/\varepsilon_{c}^{\prime }\right)}$	$k=1.05E_{cm}\varepsilon_{c}^{\prime }/f_{c}^{\prime }$ $E_{cm}=22(\frac{f_{c}^{\prime }}{10}{)}^{0.3}\varphi_{cm}^{rec}$ $\varepsilon_{c}^{\prime }=0.7{f_{c}^{\prime }}^{0.31}\alpha_{c}^{rec}$, $\varepsilon_{cu}=0.0035\beta_{c}^{rec}$

## Data Availability

Not applicable.

## Notation

*The symbols used in this paper:*

*a*, *b*	Parameters related to ascending and descending portions in the model
$E_{cm}$	Secant modulus at 0.4$f_{c}^{\prime }$
$E_{0}$	Secant modulus at $f_{c}^{\prime }$
$E_{c}$	Elastic modulus
$f_{c}$	Compressive stress
$f_{c}^{\prime }$	Peak compressive stress
$f_{max0}$	Peak compressive stress of normal concrete
$f_{28}$	Compressive strength after 28 days
k	The parameter in a model by EU code [48] and model by Belen et al. [38]
$k_{e}$	A coefficient used in proposed model
$m_{1}$	The parameters in the model by Xiao et al. [54]
*m*, *n*, *p*, *q*	The constant parameters in the model by Xiao et al. [54]
r	Volume replacement rate
t	Curing time
μ, ν	The constant parameters in the model by Xiao et al. [54]
$\alpha_{c}$	Shape parameter $\alpha_{c}$ used in Xiao et al. [54]
$\alpha_{c}^{rec}\;\beta_{c}^{rec}$	The adjustment factors in the model by Belen et al. [38]
β $,\;\beta_{m}$	A concrete material parameter
$\varepsilon_{c}$	Axial compressive strain
$\varepsilon_{c}^{\prime }$	Peak axial strain at $f_{c}^{\prime }$
$\varepsilon_{cu}$	Ultimate axial strain
$\rho_{c}$	The parameters in the model by Xiao et al. [54]
$\varphi_{cm}^{rec}$	The adjustment in the model by Belen et al. [38]

*Abbreviations:*

AAE	Average absolute error
DIC	Digital imagine collection
FRP	Fiber-reinforced polymer
GFRP	Glass fiber-reinforced polymer
HSG	Hooped strain gauge
ITZ	Interfacial transition zone
LSG	Longitudinal strain gauge
MSE	Mean square error
NAC	Natural aggregate concrete
NCA	Natural coarse aggregates
OPC	Ordinary Portland cement
RAC	Recycled aggregate concrete
RFA	Recycled FRP aggregates
RFAC	Recycled FRP aggregates concrete
RGFA	Recycled GFRP aggregates
SD	Standard deviation
